# Current Knowledge of Microglia in Traumatic Spinal Cord Injury

**DOI:** 10.3389/fneur.2021.796704

**Published:** 2022-01-11

**Authors:** Lintao Xu, Jingyu Wang, Yueming Ding, Linlin Wang, Yong-Jian Zhu

**Affiliations:** ^1^Department of Neurosurgery, Second Affiliated Hospital of Zhejiang University School of Medicine, Hangzhou, China; ^2^School of Medicine, Zhejiang University City College, Hangzhou, China; ^3^Department of Basic Medicine Sciences, and Department of Orthopaedics of Sir Run Run Shaw Hospital, Zhejiang University School of Medicine, Hangzhou, China

**Keywords:** microglia, spinal cord injury, neuroinflammation, neuron, therapy

## Abstract

Microglia are the resident immune cells in the central nervous system (CNS). After traumatic spinal cord injury (SCI), microglia undergo activation, proliferation, and changes in gene and protein expression and morphology, with detrimental and beneficial effects. Activated microglia cause secondary neuronal injury *via* the production of proinflammatory cytokines, reactive oxygen species, and proteases. However, activated microglia also promote neuronal repair through the secretion of anti-inflammatory growth factors and cytokines. Proinflammatory cytokines increase endothelial permeability, promote A1 astrocyte activation and axonal demyelination, and reduce neural stem/progenitor cells (NSPCs), leading to the exacerbation of neuronal injury. In contrast, anti-inflammatory factors facilitate angiogenesis, reduce reactive astrocytes, and promote axonal remyelination and the propagation of NSPCs, contributing to tissue repair and locomotor recovery. Due to its limited regenerative capacity, the CNS requires beneficial microglia for continuous protection against injury. Understanding and regulating microglial activation status are beneficial to reducing detrimental effects and promoting repair behaviors and to obtain more information on efficient therapies for traumatic SCI. This review discusses microglial activation and the differences between microglia and similar immune cells, microglial interactions with other cells in the spinal cord, and the progress in the development of therapies targeting microglia in SCI.

## Introduction

Microglia are resident immune cells in the central nervous system (CNS). These cells were distinguished from astrocytes ~100 years ago (1). They are important in the proliferation and differentiation of neurons and have two basic functions, immune defense, and maintenance of nervous system homeostasis (2), which includes phagocytosis of neuron debris (3), synaptic pruning (4), pruning of axonal collaterals (5), stimulation of axonal regrowth (6), and axon remyelination (7).

Microglial progenitors enter the developing brain early in embryonic development (8, 9), and the fact that microglia are derived from the embryonic yolk sac was established by cell lineage tracing (10, 11). Microglia arise from embryonic primitive hematopoietic precursors in the developing CNS by 9.5 days postconception and before the onset of bone marrow hematopoiesis (2). Thereafter, microglia are maintained by local self-renewal throughout life independent of hematopoietic stem cells (12). Recent studies have revealed that IRF8 and tumor growth factor-β (TGF-β) are indispensable molecules in microglial development (11, 13). After their development, microglia monitor the cellular environment with their ramified processes and switch their state rapidly from monitoring to protecting the injured site (14).

Traumatic spinal cord injury (SCI) is a severe event with serious effects on both the physical and psychological fitness of sufferers, which lead to a high social and economic burden on society (15). Injured axons are difficult to regenerate in the SCI environment, which results in sensory sensitivity changes, motor deficits, and autonomic dysfunction. SCI causes primary vascular damage and initiates a cascade of proinflammatory molecules and free radicals. This cascade leads to secondary damage characterized by persistent inflammation and subsequent tissue damage (16). Proinflammatory cytokines, chemokines, microglial activation, and leukocyte invasion are the dominant factors of secondary injury (17). An increasing number of studies have demonstrated that activated microglia play a vital role in the reaction to SCI by secreting proinflammatory cytokines, phagocytosing tissue debris, and promoting the resumption of tissue homeostasis.

In recent years, a large number of exploratory studies have been performed on nerve repair treatment in SCI, and some of these studies have benefited patients (18–21). Most studies have modulated the microglial phenotype to improve neuropathic outcomes. In addition, the focus has also been placed on reducing secondary injury caused by inflammation following injury. A better understanding of the roles of microglia in the response to SCI will provide insight into the progress of new therapeutic strategies to facilitate tissue recovery.

## Phenotypes of Microglia and Distinction With Macrophages

**Figure 1:**
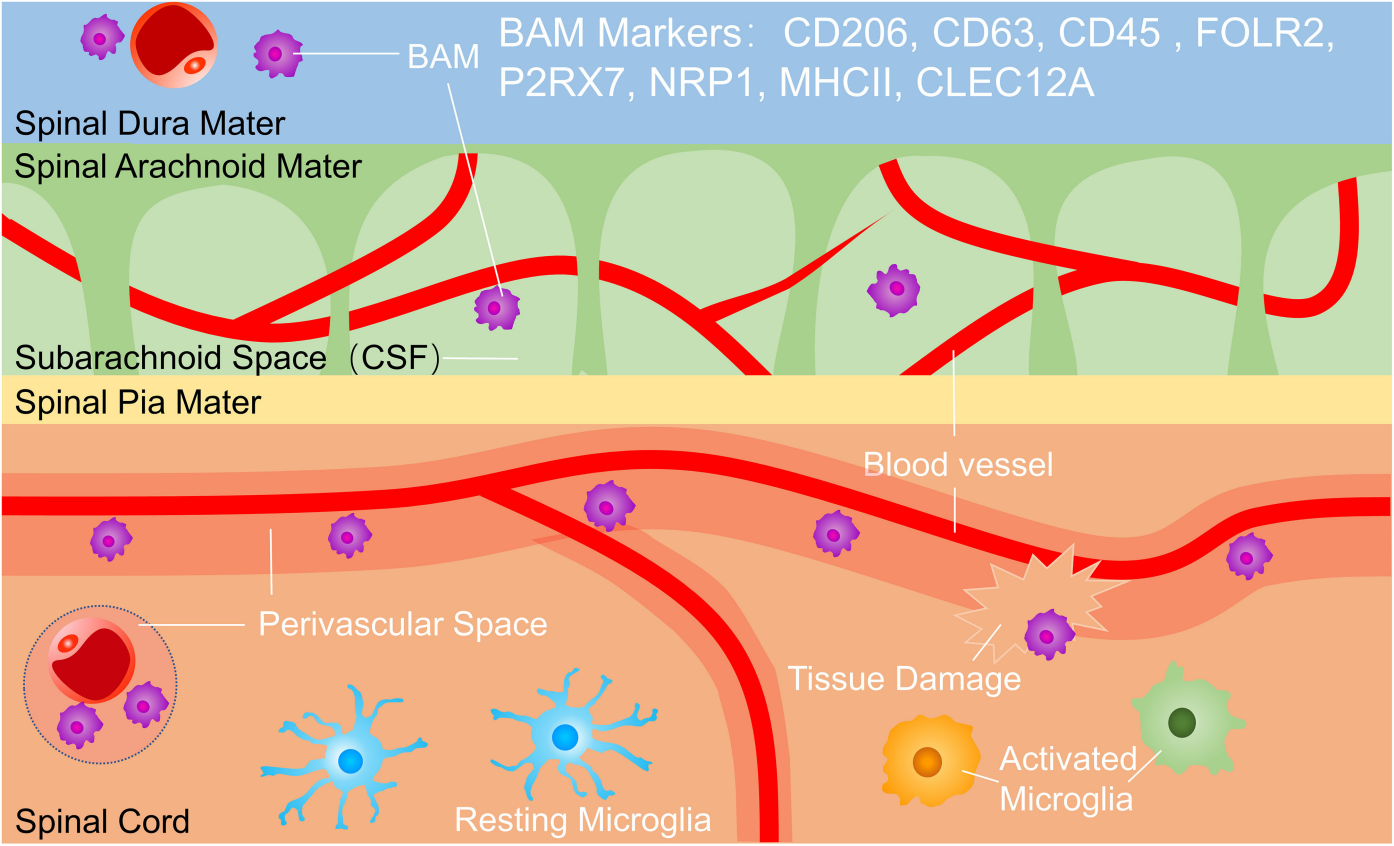
Microglia and border-associated macrophages (BAMs). BAMs reside in the dura, subarachnoid space and perivascular space. BAM markers include CD206, CD63, CD45, FOLR2, P2RX7, NRP1, MHC II, and CLEC12A, and BAMs function in immunological surveillance, phagocytosing debris, presenting antigens, and controlling the exchange of cells and cytokines between the central nervous system (CNS) and bloodstream. BAMs, border-associated macrophages.

Microglia is the only resident phagocyte in the parenchyma in the steady-state (10, 22), but microglia is easily confused with macrophages outside parenchyma termed border-associated macrophages (BAMs) which reside in the perivascular space, meninges, and choroid plexus (13, 23) (Figure 1). Microglia and BAMs are difficult to distinguish due to their similar expression of numerous classical markers, such as CD11b, the ionized calcium-binding adaptor molecule Iba-1, the fractalkine receptor (Cx3cr1), MER proto-oncogene tyrosine-protein kinase (MerTK), and the colony-stimulating factor 1 receptor (Csf1r, CD115) (23–25). Therefore, experimental approaches using Cx3cr1^GFP^, Cx3cr1^Cre^, or Cx3cr1^CreERT2^ lines usually assessed a mixture of all CNS macrophages rather than pure microglia populations. However, homeostatic microglia can be distinguished with BAMs for lower expression of CD45. Owe to Recent scRNA-seq studies, microglia have been separated from BAMs for distinct transcriptomic signatures, such as P2Y purinergic receptor (P2yr) 12, solute carrier family 2 member 5 (SLC2A5), transmembrane protein (Tmem) 119, and beta-hexosaminidase subunit beta (Hexb), whereas BAMs were enriched for membrane-spanning 4 domains subfamily A member 7 (Ms4a7), mannose receptor (Mrc) 1, and others (25, 26). These distinct transcriptional profiles suggest diverse and non-redundant functions of BAMs during health and disease. As possible gatekeepers of CNS, BAMs control the entrance of leukocytes from blood and cerebrospinal fluid into the parenchyma. BAMs also restrict the exchange of various cytokines or chemokines between CNS and blood (27) (Figure 1). The respective functions and interaction of BAMs and microglia in CNS development, homeostasis, and pathology can be studied in future research.

**Figure 2:**
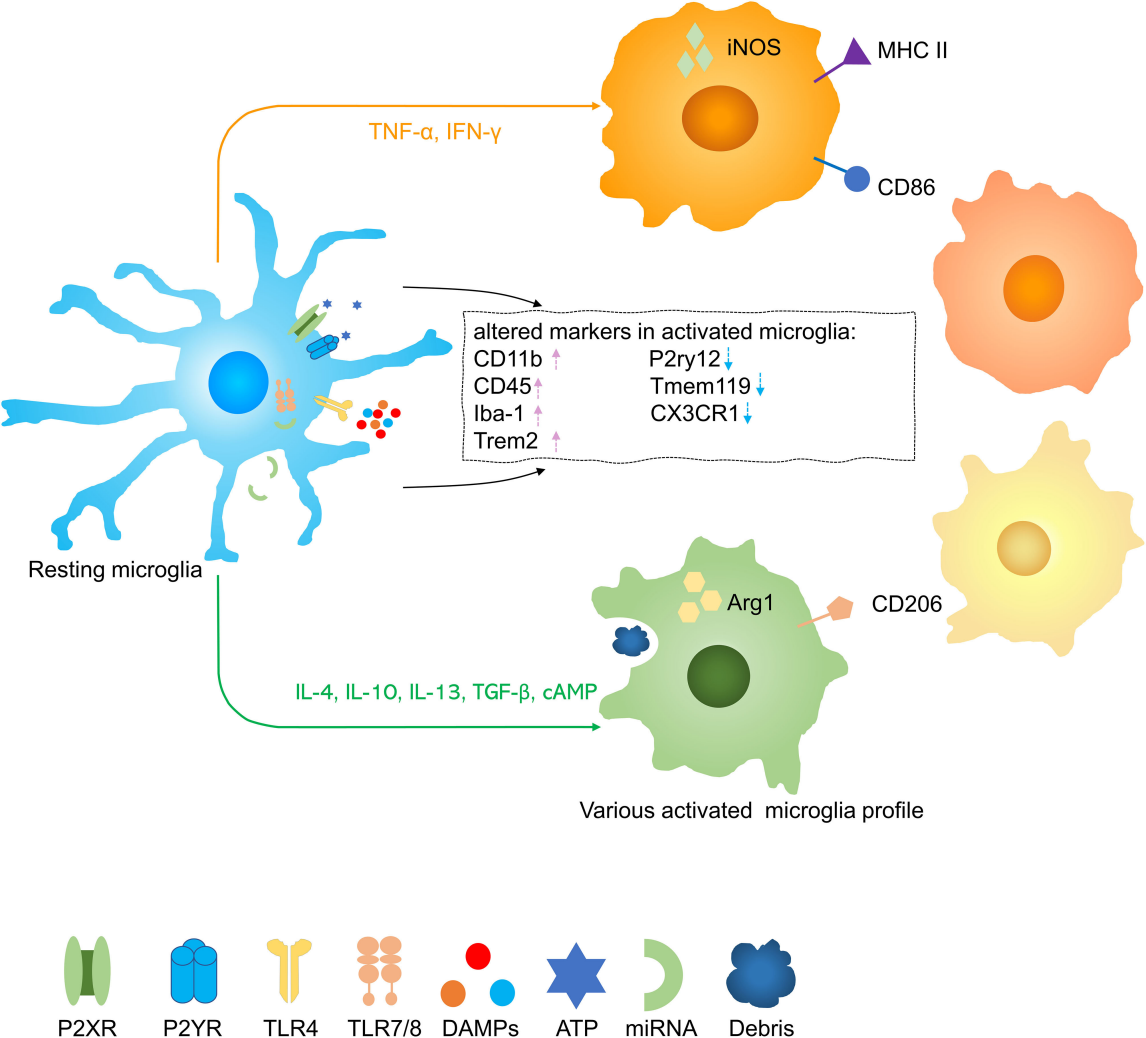
Activation of microglia in spinal cord injury (SCI). Resting microglia are activated into several phenotypes by various stimuli. Pink arrow, upregulation; blue arrow, downregulation.

Homeostatic microglia were the predominant phenotype in the uninjured spinal cord but after traumatic SCI, microglia were activated into some damage associated microglia or dividing microglia (28, 29) (Figure 2). Upon activation, microglia increase expression of CD11b, CD45, Iba-1, and Trem2 and decrease expression of P2ry12, Tmem119, and CX3CR1 (30, 31). It is more difficult to distinguish between microglia and invading macrophages after SCI. Although numerous microglia core genes were substantially downregulated during pathologies, Hexb still acts as a stably expressed microglia core gene (32). To distinguish microglia from mononuclear-derived macrophages (MDMs), a few tools were created, such as Cx3cr1^creER^::R26-TdT mice, due to slow turnover of microglia, at one month after tamoxifen treatment, nearly all (99.6 ± 0.2%) CD11b + cells in the spinal cord parenchyma expressed TdT with only a few CD11b + cells in the blood (3.8% ± 1.7%), spleen (6.7% ± 1.6%), and bone marrow (2.4% ± 0.2%) were TdT + (33). In lysozyme M^EGFP^ mice, EGFP is expressed in myelomonocytic cells but not in resting microglia (34). Some researchers transplant C57/BL bone marrow to CX3CR1^GFP^ mice which are radiated and transplant CX3CR1^GFP^ mice bone marrow to radiated wild-type mice, so they can distinguish resident microglia with blood-derived monocytes and macrophages (35). Studies have shown that infiltrated macrophages increased rapidly in the injured spinal cord around 3 days in acute stages and reach peaks at 7 days postinjury. Infiltrated macrophages decreased constantly in subacute and chronic stages, up to 42 days postinjury, it was almost 30 times less than the 3 days postinjury (36). It was figured out that MDMs move faster and are more responsible for secondary axon damage than resident microglia. This phenomenon possibly can be interpreted that infiltrative macrophages persist much more time in phagocytosing material (34). Microglia and infiltrative macrophages directly communicate with each other and distinctively modulate another's functions. Infiltrating macrophages also suppress the proinflammatory and phagocytosis functions of microglia (37). It was demonstrated that the lesion site was mainly occupied with blood-derived neutrophils and MDMs rather than microglia. The distinct functions of microglia and macrophages and their interactions still need to be further explored.

## Roles of Microglia in SCI

Microglia are very sensitive activation due to surrounding environment alternation and change their morphology rapidly to round and release of intracellular functional molecules, acting as a first active shield in CNS (38). Following a moderate contusive SCI, only 33% of microglia were left at the lesion epicenter at 1 dpi, suggesting that they underwent rapid cell death. Half of the microglia at the lesion epicenter proliferate at 4 dpi which peaks at 7 dpi. The number of microglia continued to increase at the lesion epicenter over time, reaching a peak at 14 dpi, almost 11 times more than the uninjured state (33).

Phagocytosis of cell debris is a prerequisite for recovery after injury. Expression levels of CD68, a lysosome-associated glycoprotein, a marker of phagocytosis, remained low in microglia at 1 dpi but upregulated strongly at 4 dpi, which points to a potential increase in their phagocytic activity (33). Microglia started to gradually decrease their expression of CD68 and increase their expression of P2ry12 from day 14 up to day 35, suggesting a partial return to homeostasis (33). Studies have shown that microglia come to contact with injured axons early (24 h post-SCI) but macrophage phagocyte debris 3 days later. It is worth noting that the phagocytosed debris persists in macrophages for the entire observation period (42 days) with only transient detection in microglia. Reasons for this could be disproportional cell death in microglia or more efficient processing of the phagocytosed material in microglia. Cell death after phagocytosis of myelin and cell debris was by far more frequent in MDMs compared to microglial cells both *in vitro* and *in vivo*, thereby suggesting more efficient processing of phagocytosed material as a better explanation.

**Figure 3:**
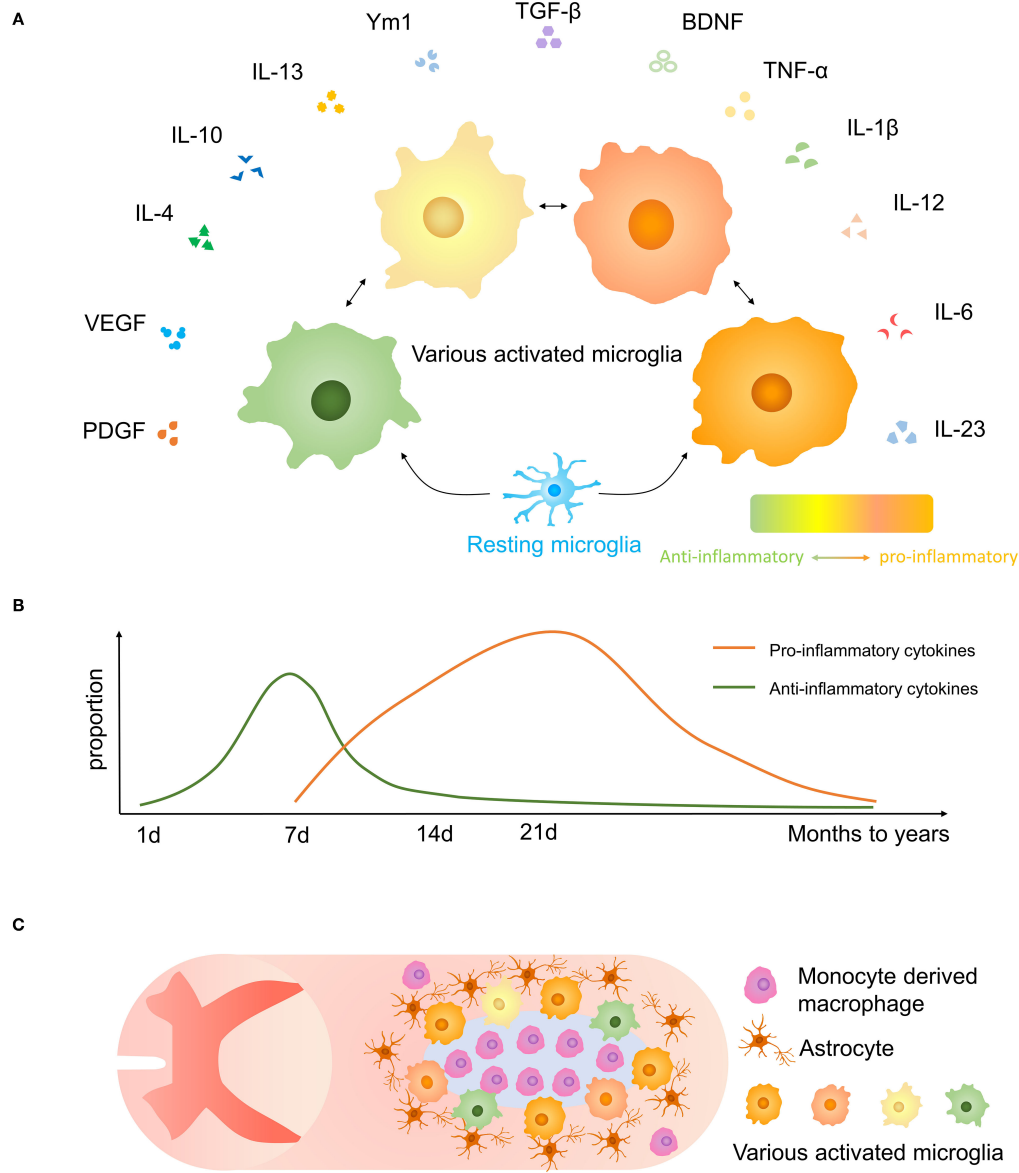
Microglial polarization after traumatic SCI. **(A)** Secretory functions of various activated microglia. **(B)** Anti-inflammatory molecules are prominent at 1-week post-injury, and proinflammatory effects are increased after 1 week and persist for months to years. **(C)** The distribution of microglia and other cells and glial scar in the injured spinal cord.

According to previous studies, activated microglia have various phenotypes reacting to inflammation which may have neurotoxic or neuroprotective effects, and they may be altered by external stimuli (39, 40) (Figures 3A,B). As far as previously known, the neurotoxic/neuroprotective effect of each microglia is not separated.

Microglia, astrocytes, together with oligodendrocyte precursor expressing neuron/glia antigen 2 (NG2) cells form a dense boundary structure called a glial scar near the injured tissue, sealing and isolating the whole injured area (41) (Figure 3C). This barrier also facilitates debris clearing, contains inflammatory cytokines, and promotes wound compaction and matrix reorganization to minimize the scar size and maximize the repair area (42). After SCI, microglia markedly increased expression of Peroxiredoxin 1/proliferating cell nuclear antigen, and they play a vital role in astrocyte and microglia proliferation (43). Besides, microglia are important in inducing the formation of an astrocytic scar as it secrets cytokines, such as insulin-like growth factor-1 (IGF-1), to facilitate propagation and activation of astrocytes (33). Activated microglia move to the interval between surrounding astrocytes and central infiltrating immune cells. Using CSF1R inhibitor PLX5622 which has the potential to cross the blood–spinal cord barrier to delete microglia, resulted in the destruction of the glial scar, which leads to the aggravation of immune cell infiltration, extensive spread of inflammation, and poor locomotor outcomes (33). And the result was consistent in another experiment (44). Recently, Zhou et al. demonstrated that Plexin-B2 in injured-activated microglia protected the structure of the astrocyte barrier and promoted corralling and wound compaction (42). In neonatal mice, a crush SCI leads to microglia-organized scar-free repair that allows the growth of long projecting axons through the lesion (28). Besides, both neonatal microglia and adult microglia treated with peptidase inhibitors obviously enhanced tissue repair and axon regrowth. Microglia in neonatal mice facilitated this healing process by secreting fibronectin and its binding proteins to bond the broken ends of the spinal cord and expressing several peptidase inhibitors to resolve inflammation (28).

However, glial scar also acts as a significant obstruction for neuronal regeneration after SCI. Glial scar and its secreted cytotoxic cytokines, such as chondroitin sulfate proteoglycans (CSPGs), negatively affect nerve function regeneration after SCI. The formation of a physical barrier and some inhibition signals lead to axon regeneration disturbance and remyelination disturbance of regenerated axons (45). Yet not all CSPGs showed an inhibitory effect on nerve regeneration. For example, studies have shown that CSPG4 (NG2) and CSPG5 (also known as neuroglycan C) have functions promoting axonal growth (46, 47). The specific role of glial scar in various periods remains to be studied.

Microglia has abundant interactions with other resident cells in SCI, including neurons, astrocytes, oligodendrocytes, neural stem cells, and endothelial cells. Distinct phenotypes of microglia have respective effects on those cells.

### Effect of Microglia on Neurons

In the resting state, microglia play the role of immune surveillance, maintain the stability of the environment and regulate neural circuit development in the immature CNS (48). After SCI, microglia phagocyte axon fragments and secret pro/anti-inflammatory molecules affect neurons regeneration. Persistent microglia activation with chronic neuroinflammatory appears after CNS injury and it contributed to following neurodegeneration and neurological deficits. Scars consisting of microglia can isolate axons from cytotoxic immune cells in core lesions of SCI and deletion of microglia is proved to be disadvantageous to neurons regeneration (33). Fibroblast growth factor 10 (FGF10) derived from neuron and microglia/macrophage increased after SCI and it reduces tissue injury and neuron loss, enhances axonal sprouting, facilitates tissue repair, and promotes functional recovery from SCI *via* FGFR2/PI3K/Akt pathway (49).

One study indicated that microglia elimination essentially did not influence neuronal repair after retinal ganglion cell (RGC) injury (50). Although the environment and function of microglia in RGC and CNS are not consistent, Willis et al. demonstrated that sustained depletion of microglia has a slight effect on neurogenesis after CNS injury (51). And they figured out that turnover of microglia to a neuroprotective phenotype stimulated functional neurogenesis after TBI, these repopulating microglia took neuroprotective effect which is highly dependent on the appropriate time window in the acute phase of injury. Consistent with this, another study suggested that fewer neurons were retained in the spinal cord lesion area in the microglia depletion group compared with the vehicle group at 35 days postinjury (33).

In another experiment, adult male C57BL/6J mice were treated with PLX5622 to remove chronically activated microglia one month after CNS injury and discontinued one week later to allow microglia to regenerate (52). The repopulated microglia showed branching morphology similar to sham-operated mice, while the microglia in-vehicle CNS injury mice showed a typical amoeboid morphology in the chronic posttraumatic state. Mice treated with PLX5622 lead to fewer neuropathological changes, such as limited cortical injury, less neuron cell death, and reduced NOX2 and NLRP3 associated neuroinflammation. Therefore, short-run depletion of activated microglia in the chronic phase of CNS injury followed by regeneration leads to the long-term recovery of neurological function, suppression of oxidative stress pathways, and neuroinflammation, with a reduction in persistent neurodegenerative processes. Therefore, microglia influence neurons regeneration based on their phenotype and action time. The effects of activated microglia and repopulating microglia in different stages on neurons need to be further investigated.

The relationship between microglia and neurons is closer than expected. It is found that the microglia-neuron interface exists in physical interactions. Microglia immune surveillance is regulated by excitatory and inhibitory neurotransmission. Microglia have been demonstrated to modulate neurotransmission and selective activation of microglia enhances excitatory neurotransmission (53, 54). However, the contact between microglia and damaged axons does not necessarily lead to phagocytosis, but may also enhance regeneration through physical contact, which can greatly change the current cognition of neuroinflammation and promote the progress of new treatments for axonal injury (55). Cserép et al. found that a specialized nanoarchitecture communicated by purinergic signaling exists in somatic microglia-neuron junctions (56). The microglial processes at these junctions can potentially surveil and protect neuronal function as changes in the somatic junction give rise to P2Y12 receptor-dependent neuroprotection, alter functional connectivity, modulate neuronal calcium load, and protect neuronal functions (56).

Nerve injury often causes suffering chronic pain, known as neuropathic pain, which is resistant to treatment with currently available painkillers (57). It is known that neuropathic pain is closely related to severe neuroinflammation, in which proinflammatory cytokines and chemokines play a crucial role in the progress and persistence of neuropathic pain (58). As various matches of ligand and receptor exist in glial cells (microglia and astrocytes) and neurons, chemokines can play a role in inducing glial activation or promoting the excitatory synaptic transmission of spinal neurons which amplifies central sensitization (58). A study has shown that spinal blockade of microglia P2X4R and CX3CR1 signaling can reduce early state mechanical allodynia of nerve injury (59). And blocking the chemokine receptors of astrocytes, such as CXCR2, CXCR3, CXCR5, and CCR2, may relieve late state neuropathic pain (58). Interactions between microglia and injured neurons are critical for the progression of chronic inflammation and neurological improvement after SCI.

### Effect of Microglia on Astrocytes

**Figure 4:**
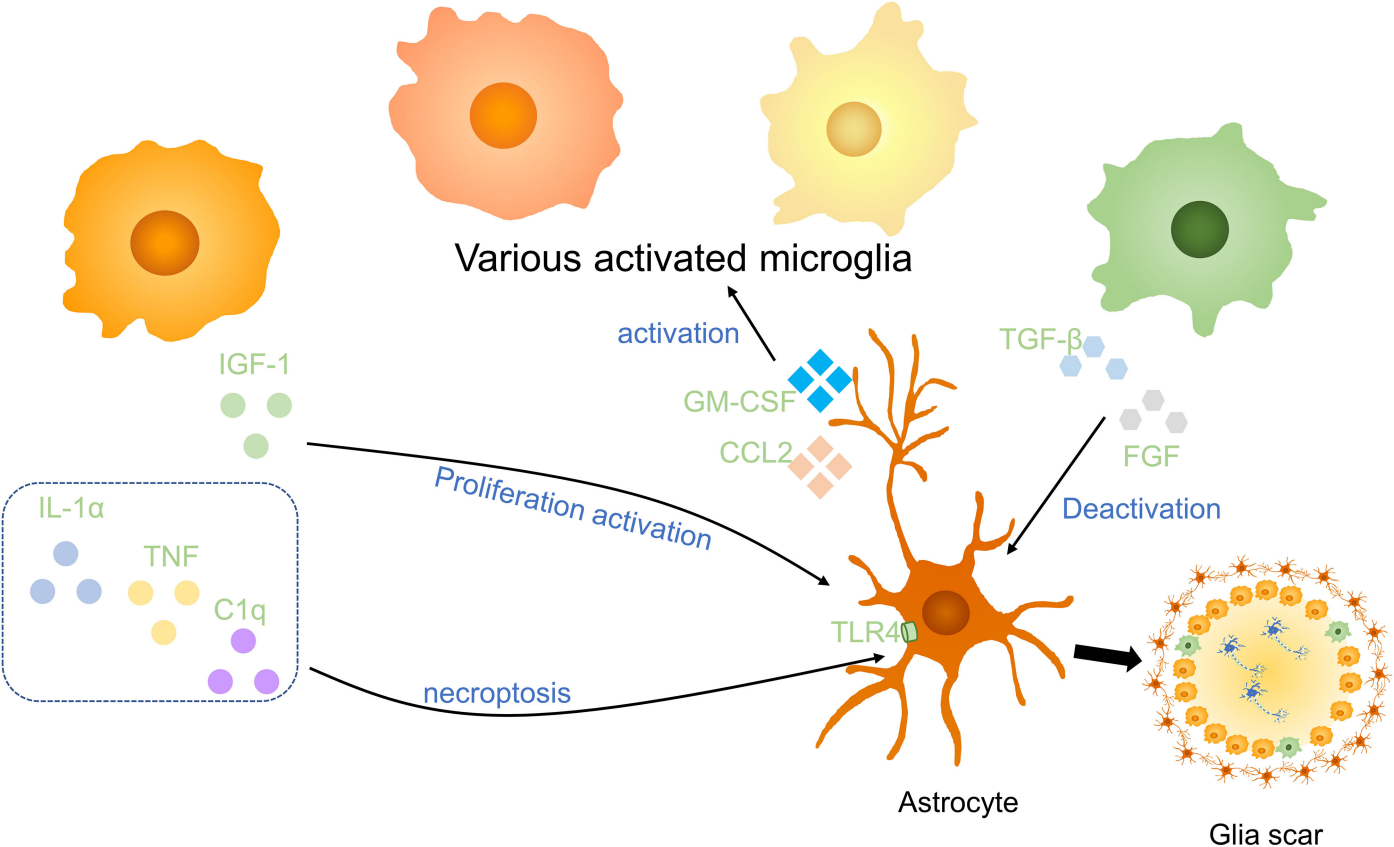
Effects of microglia on astrocytes in SCI. Anti-inflammatory cytokines including insulin-like growth factor-1 (IGF-1) and the combined effect of IL-1α, TNF, and C1q promote the activation and proliferation of astrocytes. Anti-inflammatory factors, such as TGF-β and FGF, to deactivate astrocytes. Astrocytes activate microglia *via* GM-CSF and CCL2. Activated microglia and astrocytes are essential parts of glial scars in the injured spinal cord.

Microglia are associated closely with astrocytes, which have numerous stellate processes in a healthy state, they react rapidly to alternation of environment and release molecules to modulate astrocytes (Figure 4). After SCI, microglia stimulate the proliferation and activation of astrocytes to produce a variety of SCI outcomes. Using Csf1r^−/−^ knockout mice which wipe out microglia, Liddelow et al. reported that classically activated microglia secret IL-1α, TNF, and subcomponent q(C1q) to induce neurotoxic astrocytes. These reactive cells are named A1 astrocytes. A1 astrocytes lose normal astrocyte functions, such as preventing neuronal death, facilitating axons sprouting, synaptogenesis, and phagocytosis of synapses and myelin debris. On the contrary, it gave rise to a powerfully neurotoxic effect of killing neurons and induce the decrease of mature oligodendrocytes (60). Mice treated with pexidartinib (PLX-3397), a colony-stimulating factor 1 receptor (CSF1R) inhibitor, resulting in 95% depletion of microglia still produce A1 astrocyte as a response to lipopolysaccharide (LPS). However, Csf1r^−/−^ knockout mice that wipe out microglia fail to produce A1 astrocytes. It indicated that a small part of microglia is enough to activate A1 astrocytes. IL-1α, TNF, and C1q are the strongest inducers of A1 astrocytes which are increased in LPS-activated microglia-conditioned medium. Pretreated with IL-1α, TNF, and C1q neutralizing antibodies, LPS-activated microglia-conditioned medium failed to induce A1 astrocytes. It is worth noting that triple-knockout (IL1a^−/−^Tnf^−/−^C1qa^−/−^), double-knockout (IL1a^−/−^Tnf^−/−^), and single-knockout (IL1a^−/−^, Tnf^−/−^, C1qa^−/−^) mice in which microglia still secreted inflammatory cytokines was unable to induce A1 astrocytes after LPS injection, suggesting that IL-1α, TNF, and C1q are not only sufficient but also necessary cytokines to induce A1 astrocytes (60). However, following depletion of IL-1α, TNF, and C1q could not revert astrocyte phenomenon, anti-inflammatory cytokine TGF-β and FGF have been proved to decrease A1 astrocyte transcript levels *in vitro* (60).

Consistent with this, Kang et al. indicated that FGF signaling activation decreases the response of astrocytes and promotes their deactivation after CNS injury (61). Through astrocyte-specific P2Y1 receptor overexpression and knockdown, studies have demonstrated that neuroprotective A2 astrocytes are induced by microglia to support the growth of neurons *via* downregulation of the P2Y1 purinergic receptor (62). In addition, astrocytes also enhanced microglial activation and boosted microglial TNF-α production in brain inflammation by producing proinflammatory molecules, for instance, C-C motif chemokine ligand 2 (CCL2) and granulocyte-macrophage colony-stimulating factor (GM-CSF) (63).

Microglia are vital in astrocyte proliferation and glial scar formation. In addition, a study demonstrated proinflammatory microglia also induce necroptosis of cavity-surrounding reactive astrocytes partially *via* TLR/MyD88 (myeloid differentiation factor88) signaling, and SCI secondary damage may be weakened by inhibition of astrocytic necroptosis (16). The above information indicates that microglia have effects on astrocytes both promoting its proliferation and necroptosis, and its various functions still need to be illuminated. Synergized with reactive astrocytes, microglia segregate infiltrating immune cells at the center of the lesion (33). Glial scar formation has the ability to alleviate neuron degeneration at early stages post–SCI (33, 64). Therefore, in future work, it will be of great interest to explore the interactions between microglia and astrocytes, with their effects on damage repair, in animal models of SCI using cell-specific strategies.

### Effect of Microglia on Oligodendrocytes and Oligodendrocyte Progenitor Cells

**Figure 5:**
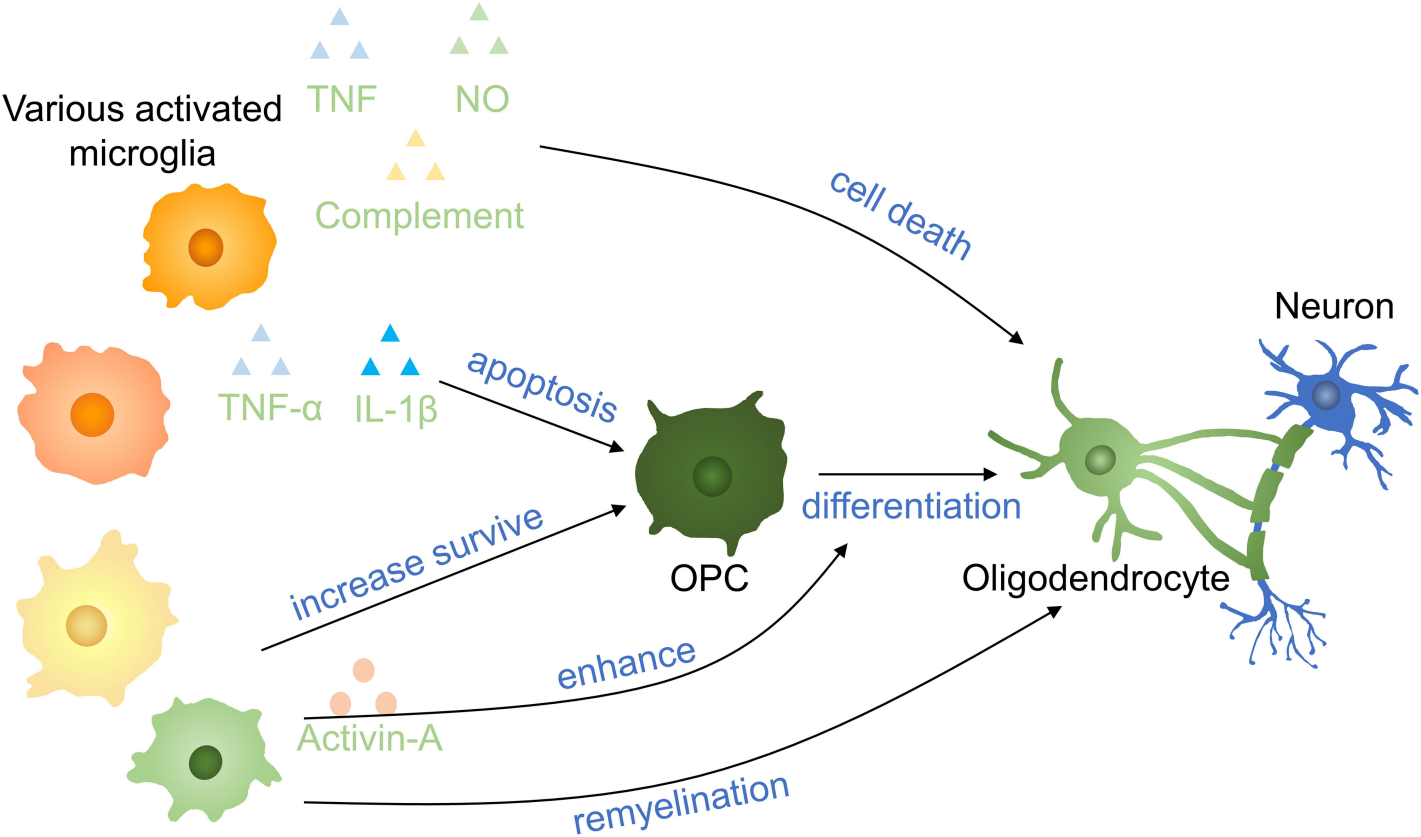
Effects of microglia on oligodendrocytes and OPCs in SCI. Proinflammatory factors induce OPC apoptosis and oligodendrocyte death. Anti-inflammatory cytokinnes increase the survival of OPCs and promote the remyelination of neurons. Activin-A (a TGF-β superfamily member) derived from activated microglia enhances the differentiation of OPCs into oligodendrocytes. OPCs, oligodendrocyte precursor cells.

Microglia regulate oligodendrocyte survival and function in CNS development and it is helpful for normal myelinogenesis and oligodendrocyte progenitor cells (OPCs) maintenance during adulthood in healthy individuals (65). Axon demyelination and oligodendrocyte cell death take place after SCI, and it is important in secondary injuries leading to persistent neurodegeneration in SCI (66). Microglia are either beneficial or noxious in different pathological contexts to oligodendrocytes (67) (Figure 5). Activated microglia secreting TNF, NO, and complement to promote oligodendrocyte cell death and phagocytosis by microglia (68, 69). Microglia activation is induced by S100A8/A9 which activates microglia and switch microglia to secreting more inflammatory molecules, facilitating the apoptosis of OPCs *via* activating the NF-κB signaling pathway (70). On the other hand, oligodendrocyte cell death decreased 5 days postinjury in mice after intraperitoneally injected with fluoxetine partially by inhibiting microglia activation (66). Supernatants from proinflammatory, but not anti-inflammatory, microglia have a deleterious influence on human A2B5 + neural progenitors, leading to decreased oligodendrocyte cells and an indirect effect on OPCs differentiation by promoting astrocyte-derived CXCL10 *in vitro* (71). In a toxic model of oligodendrocyte demyelination, it was found that decreased neuroinflammation induced by microglia reduction enhanced central remyelination (72).

A gradual transformation of microglia from proinflammatory to anti-inflammatory state occurred with oligodendrocyte cells remyelination start. Anti-inflammatory microglia conditioned media prevented OPC apoptosis and enhanced OPCs differentiated to oligodendrocyte by activin-A *in vitro* and differentiation was impaired following anti-inflammatory microglia depletion (73). In the presence of the extracellular matrix (ECM) protein laminin, microglia secreted transglutaminase-2 drives OPCs proliferation and myelination *via* adhesion G protein-coupled receptor G1 signaling. Bellver-Landete et al. have shown that compared with the vehicle group at 35 days postinjury, fewer oligodendrocytes were seen in the lesion site of the spinal cord in the PLX5622 group, improving that microglia are essential for oligodendrocytes survival post-SCI (33). The possible explanation may be that the overall effect of anti-inflammatory molecules is more obvious than inflammatory molecules secreting by microglia in the first month. This may be also an interpretation for different outcomes of Beckmann and Victor (33, 72). In recent research, microglia is proved to facilitate oligodendrogenesis after CNS injury (74). Therefore, various microglia profiles have entirely variant impacts on oligodendrocytes and OPCs, and the specific mechanism needs to be further clarified.

### Effect of Microglia on Neural Stem/Progenitor Cells

**Figure 6:**
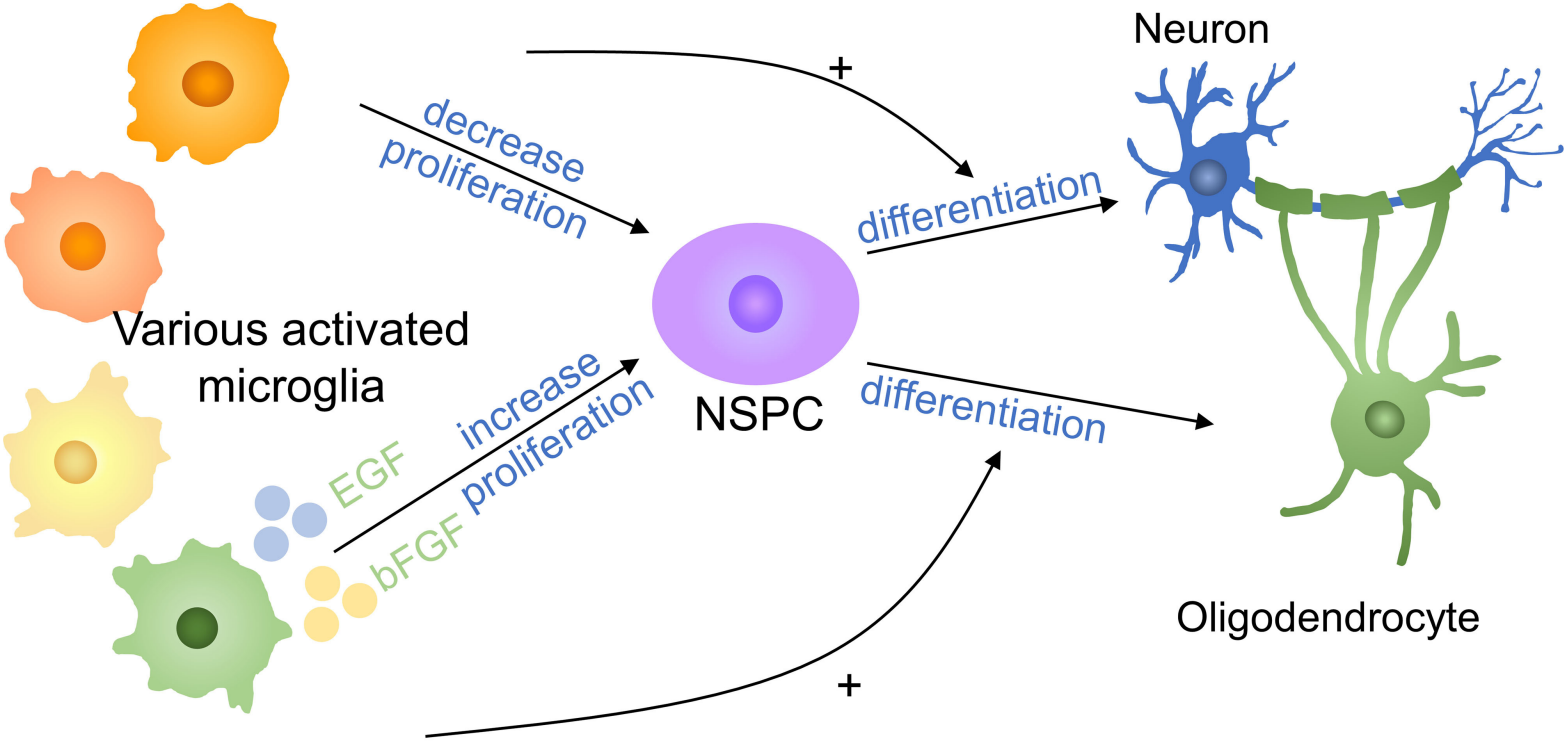
Effects of microglia on NSPCs in SCI. Proinflammatory factors decrease the proliferation of NSPCs, whereas anti-inflammatory cytokines increase NSPC proliferation. Proinflammatory factors promote NSPC differentiation into neurons, whereas anti-inflammatory molecules promote NSPC differentiation into oligodendrocytes. + means promotion. NSPC, neural stem/progenitor cell.

Neural stem/progenitor cells (NSPCs) neurogenesis occurs in CNS and NSPCs have the potential to duplicate and differentiate to neurons, oligodendrocytes, and astrocytes and are important in spinal cord repair (75–77). It was reported that microglia stimulated by LPS or IFN-γ released cytokines to reduce NSC proliferation (78) (Figure 6). Cultured neural NSPCs with BV2-derived soluble molecules with basic fibroblast growth factor (bFGF) and epidermal growth factor (EGF), a study has shown that NSPCs had a better maintain of nestin expression and promoted proliferation compared with those cultivated with epidermal growth factor or fibroblast growth factor only. In addition, deactivated microglia conditioned medium has positive effects on NSPC proliferation and augment the population of NPCs by 50% in the immature cortex (79, 80). Therefore, non-inflammatory microglia assist neural stem cell propagation by producing neurotrophic molecules. In another study, authors put forward that microglia subtypes have a distinct impact on NSCs regulation and induce inflammatory property production with cells generation. Compared with NSCs kept in LPS-stimulated microglia conditioned medium, NSCs cultured in control microglia conditioned medium had a higher survival rate, promoted migration, and less differentiation to astrocytes (78). TNF derived from activated microglia promoted NPCs proliferation by interacting with TNF-R1 but TNF restrained neurogenesis (81). A study has demonstrated that anti-inflammatory molecules secreting by microglia supported oligodendrogenesis, on the contrary, microglial inflammatory molecules showed promotion toward neurogenesis (82). The effects of various microglia on NSPCs are still limited to a few experiments and the exact mechanism deserved further investigation.

### Effect of Microglia on Endothelial Cells

**Figure 7:**
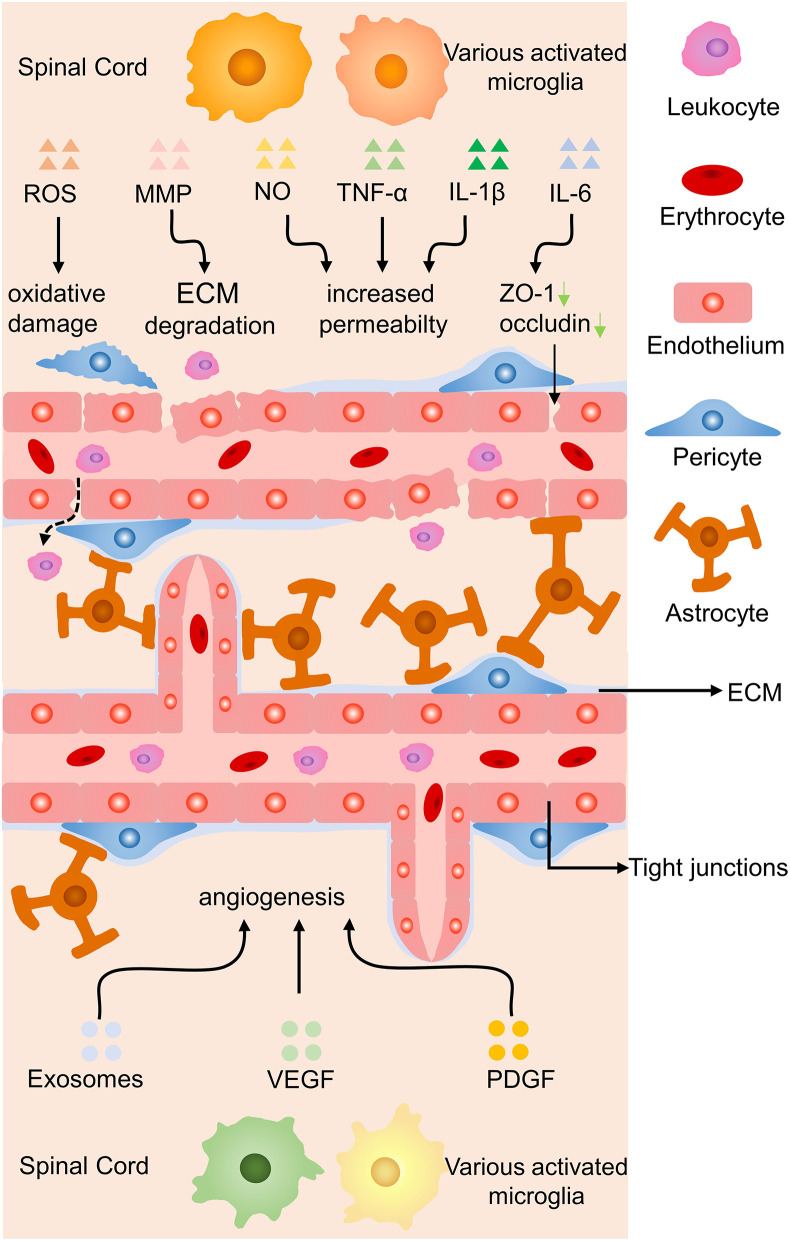
Effects of microglia on endothelial cells in SCI. Proinflammatory cytokines induce oxidative damage to endothelial cells, ECM degradation, and increased permeability of the BBB by proinflammatory molecules, while anti-inflammatory secretion promotes angiogenesis. Green arrow, downregulation.

Endothelial cells are essential components of blood-brain barrier (BBB) and blood-spinal cord barrier, special endothelial structures which selectively detach the blood circulation from the brain parenchyma. Microglia may lead to the increased permeability of the barriers, leukocytes infiltration, and angiogenesis in abnormal situations (17, 83) (Figure 7). Reactive oxygen species released by activated microglia resulting in the oxidative damage of endothelial cells, upregulation of iNOS and NO resulting in the increased permeability of BBB, together with IL-1β and TNF-α aggravate the infiltration of peripheric leukocytes into the CNS parenchyma (83, 84). Under a high glucose condition co-cultured endothelial cells with microglia, IL-6 derived from microglia led to STAT3 activation in endothelial cells, resulting in increased endothelial permeability through downregulated occludin and ZO-1 production in tight junctions (85). Microglia-derived inflammatory cytokines also have the ability to degrade ECM proteins and break BBB by producing matrix metalloproteinase (86).

Another molecular mechanism is unveiled that hypoxia-induced microglia upregulated basigin-2 expression and release IGF-1 by promotion of phosphatidylinositol 3-kinase (PI3K)-Akt pathway to induce angiogenesis (87). In a co-culture experiment, activated microglia were proved to have the ability to promote angiogenesis, migration of retinal microvascular endothelial cells, reduce the production of tight junction proteins *via* increasing the expression of platelet-derived growth factor-BB and vascular endothelial growth factor-A (88). Stroke mice treated with metformin increased anti-inflammatory cytokines secretion of microglia, facilitated angiogenesis and neurogenesis leading to promoted locomotor recovery (89). Anti-inflammatory cytokines of microglia might facilitate recovery from ischemic stroke through accelerating angiogenesis by releasing higher amounts of exosomes containing miRNA-26a (90). In brief, microglia-derived inflammatory cytokines increase the permeability of blood vessels while anti-inflammatory molecules contribute to angiogenesis by various signaling.

## Targeting Microglia in SCI Therapy

Regardless, microglia are a double-edged sword in SCI. During neuroinflammation, microglia can play a positive role in repairing the injured spinal cord but can also switch to a destructive role in which they secret excess cytotoxic cytokines and reactive oxygen mediators. Therefore, regulating the number or phenotype of activated microglia and alleviating inflammatory may provide a feasible method for treating SCI and promoting functional recovery.

### CSF1R Inhibitor

Colony-stimulating factor 1 (CSF1) modulates microglial survival, propagation, and differentiation; hence, CSF1R inhibitor treatment has the ability to remove microglia (91). After treatment with GW2580 (a CSF1R inhibitor), the number of proliferating microglia at the injury site was significantly decreased in the SCI group, while the microglial population was not changed in the non-injured spinal cord, and GW2580 treatment improved locomotor recovery in injured animals (91). To attenuate inflammation induced by activated microglia/macrophages in SCI, Ma et al. used PLX3397 (a CSF1R inhibitor) combined with photocrosslinked hydrogel transplantation to deplete activated microglia/macrophages, leading to delayed microglial repopulation. The treatment resulted in reduced CD68-positive reactive microglia/macrophages and inflammatory molecule transcript levels significantly increased differentiation of NSPCs into neurons in the lesion site and improved functional recovery compared with those of single treatment methods (92). However, studies have shown that the selective depletion of microglia by CSF1R in mice results in diffuse inflammation and an increase in lesion size or the appearance of satellite lesions after CNS injury, and this effect was reversed by microglial repopulation (33, 93). Therefore, it is important to apply this strategy within the appropriate time window, especially in the chronic state.

### Cytokine Therapy

**Figure 8:**
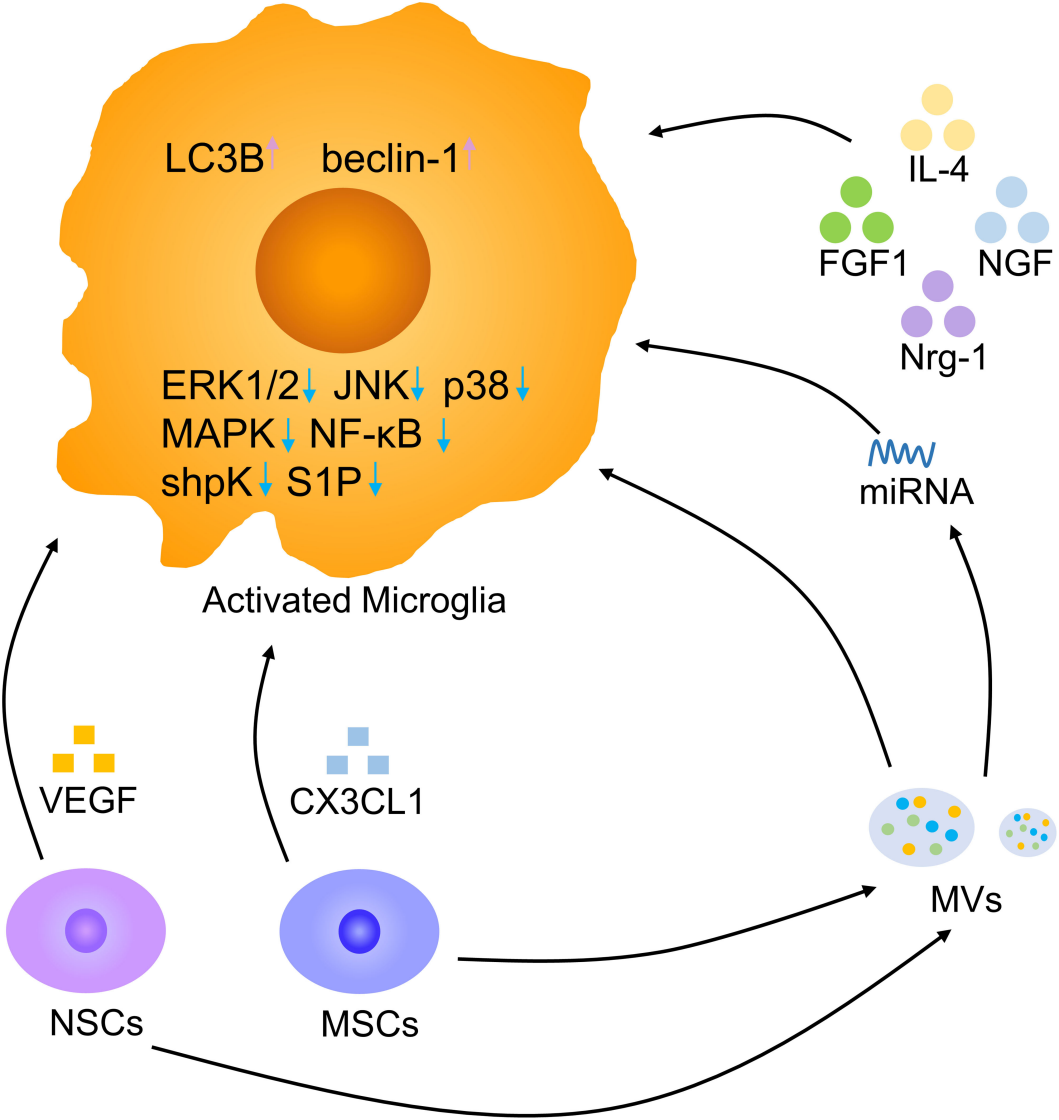
Microglia modulation by biotherapy. Multiple cytokines, NSCs, MSCs, and MVs derived from stem cells, and miRNAs derived from MVs contribute to a decrease in activated microglia. Pink arrow, upregulation; blue arrow, downregulation.

Cytokines are micromolecular polypeptides and glycoproteins with a wide range of biological activities that are synthesized and secreted by various cells. Through the secretion of cytokines, cells have the ability to communicate with other cells and perform complex multicellular activities leading to the growth, differentiation, death, and activation of cells (94). Cytokine therapy mainly includes cytokine replacement therapy and cytokine blocking therapy, and the former is the most common method and includes the use of IL-4, FGF1, and nerve growth factor (NGF) (Figure 8). Intraspinal injection of the anti-inflammatory cytokine IL-4 increased the numbers of anti-inflammatory microglia and macrophages, markedly reducing tissue injury and improving motor outcomes (95). Intravenous injection of FGF1 promoted injury recovery by inhibiting the proliferation and activation of microglia/macrophages *via* the TLR4/NF-κB pathway and also affected neurite repair through the FGFR2/PI3K/Akt pathway (49). Neuregulin-1 (Nrg-1) attenuated the response of microglia under stressful conditions by reducing the generation of the proinflammatory mediators NO, IL-1β, and TNF-α (36). NGF was shown to enhance the microglial phagocytosis of Aβ and accelerate its degradation, and NGF maintained neuronal integrity to prevent the loss of dendritic spines. The neuroprotective effects of NGF on microglia are mediated by its ability to switch them to secret anti-inflammatory molecules (96). Cytokines improve SCI recovery by altering the status of microglia. There are still numerous cytokines that need to be explored, and better drug delivery methods should be found.

### Stem Cell Transplantation

Stem cell transplantation offers feasible therapeutic methods for neurodegenerative disorders (97–99). In an Alzheimer's disease model, the transplantation of human neural stem cells (hNSCs) at the fimbria fornix improved cognitive ability after 4 and 16 weeks. The amyloid plaque load was decreased in hNSC transplantation mice by the enhanced phagocytosis of microglia (76). In mice with cortical impact injury, microglial activation was decreased in the hNSC transplantation group compared to the vehicle group. hNSC transplantation facilitated microglia to secret anti-inflammatory molecules by reduced proinflammatory INF-γ receptor β levels and promoting anti-inflammatory IL-4 receptor α expression. NSCs are beneficial to neuronal regeneration after CNS injury by modulating microglia secret anti-inflammatory cytokines (100). NSCs can release large amounts of vascular endothelial growth factor (VEGF) to inhibit microglial proliferation, migration, and phagocytosis. Removing VEGF from NSC-conditioned medium reversed these effects (101). Adipose mesenchymal stem cells (MSCs) also have immunosuppressive properties by reducing LPS-induced effects on microglial activation, such as proliferation and cytokine secretion, by sphingosine kinase/S1P signaling (102). MSCs can release CX3CL1 to modulate the response of microglia, resulting in more anti-inflammatory cytokines secretion (103). Thus, stem cell transplantation could increase microglial anti-inflammatory molecules release and alleviate neuroinflammation after SCI. Recently, it has been reported that the NSC can be better used to promote damage repair by engulfing anti-inflammatory nanoparticles (104). Exploring the effect of exogenous NSC on SCI, the way of combining new materials and targeted delivery of NSC may become a research hotspot in the future (105).

### Stem Cell-Derived Extracellular Vesicle Transplantation

Recent studies have suggested that extracellular vesicles (EVs), including microvesicles (MVs) and exosomes, are essential vehicles for paracrine signaling and play a significant role in interactions among cells to promote CNS development and repair (106, 107). The application of NSC-MVs markedly reduced microglial activation, alleviated SCI-related impairments and neuronal apoptosis, and promoted locomotor recovery at an early stage. NSC-MVs promoted the expression of LC3B and beclin-1, which are autophagy marker proteins, and increased autophagosome formation. The inhibitory impact of NSC-MV treatment on apoptosis and neuroinflammation was reduced by the application of the autophagy inhibitor 3MA. Hence, NSC-MVs decrease apoptosis and inflammatory reactions by inducing autophagy (77). MSCs can also shed MVs, which are effective regulators of microglial activation. MSC-MVs reduced the secretion of IL-1β, IL-6, and TNF-α by BV-2 cells in response to LPS *in vitro*. After co-culture with MSC-MVs, BV-2 cells expressed higher levels of the anti-inflammatory microglial marker chemokine ligand-22 by suppressing the ERK1/2, JNK, and p38 MAPK pathways (108). Regulating microglia with MSC-MVs might be a promising therapeutic method in the future. In addition, MSC-derived exosomes reduced proinflammatory gene expression of microglia by interfering with TLR4 signaling in BV-2 microglia, stabilizing the NFκB inhibitor IκBα and activating the MAPK family (109, 110). MSC-derived exosomes contain microRNAs targeting microglia and lead to the switching of microglia (111). Microglia are the major target of bone marrow MSC exosomes. Bone marrow MSC exosomes have the potential to repair SCI by inhibiting A1 reactive astrocyte activation induced by activated microglia (112, 113). Stem cell-derived EVs represent a promising future cell-free therapy to treat severe damage and lead to better outcomes. Combining new materials to increase EV effects on SCI repair is also a future trend (114).

### MicroRNAs Therapy

MicroRNAs (miRNAs), key molecules that regulate cell biological processes, are essential in the CNS due to their modulation of internal cellular signaling pathways (Table 1). Regulation of miRNAs is an alternative method to modulate gene expression and promote anti-inflammatory cytokines secretion of microglia. miRNAs are short (18–22 nucleotides), non-coding epigenetic regulatory RNAs that repress target gene expression posttranscriptionally by binding the 3'-untranslated region of target mRNAs, which results in the hydrolysis of the target mRNA (128). For example, miR-873a-5p is an essential component of exosomes derived from activated astrocytes and is highly expressed in the injured CNS. MiR-873a-5p rapidly inhibited inflammation and promoted anti-inflammatory cytokines release following CNS injury *via* the decreased activation of the ERK and NF-κB pathways (117). Transfection of miR-133b and miR-124 downregulated the expression of proinflammatory factors and reduced inflammation in CNS neurodegenerative conditions (115, 116). In addition, miR-100 and miR-183 have the potential to inhibit microglial activation and secretion of inflammatory cytokines *via* the NF-κB signaling pathway in CNS injury and ischemia (126, 127).

**Table 1:** MicroRNAs therapy.

**MicroRNA**	**Source and methodologies**	**Effects**
MiR-133b	Extraneous: lentiviral delivery	Downregulate the expression of chondroitin sulfate proteoglycans, RhoA and inhibit microglia/macrophage proliferation (115)
MiR-124	Extraneous: cell transfection	Reduced proinflammatory molecules (116)
MiR-873a-5p	Endogenous: astrocyte-derived exosomes	Reduce inflammatory cytokines, increase anti-inflammatory factors (117)
MiR-34a	Extraneous: intrathecal injection	Reduce inflammatory cytokines secretion of microglia, promote neural recovery and locomotor function (118)
MiR-150	Extraneous: cell transfection	Decrease the expression of pro-inflammatory cytokines (119)
MiR-216a-5p	Endogenous: MSC-derived exosomes	Switching microglial phenotype to secret anti-inflammatory molecules (111)
MiR-340-5p	Extraneous: cell transfection	Attenuate inflammation, oxidative stress, and apoptosis (120)
MiR-23b	Extraneous: cell transfection	Inhibited the BV-2 cells apoptosis and NF-κB activation (121)
MiR-193a antagomir	Extraneous: cell transfection	Reduced expression of neuroinflammatory molecules (122)
miR-429 inhibitors	Extraneous: cell transfection	Reduced expression of neuroinflammatory molecules (123)
MiR-223-5p inhibitor	Extraneous: intrathecal injection	Reduced expression of neuroinflammatory molecules, reduced neuron apoptosis and promoted locomotor recovery (124)
MiR27a	Extraneous: cell transfection	Alleviate apoptosis and inflammatory injury (125)
MiR100	Extraneous: cell transfection and intrathecal injection	Suppresses microglia activation and neuronal apoptosis (126)
MiR-183	Extraneous: intracerebroventrivular injection	Inhibits microglia activation and expression of inflammatory factors (127)

Quantitative real-time-PCR analysis showed that miR-23b, miR-34a, miR-150, miR-27a, and miR-340-5p were negatively regulated following SCI. MiR-23b inhibited BV-2 cell apoptosis and the NF-κB pathway (111, 119, 121, 125). MiR-34a, miR-150, miR-27a, and miR-340-5p promote microglial anti-inflammatory molecules secretion and attenuated inflammatory damage, oxidative stress, and apoptosis (120). MiR-193a, miR-429, and miR-223-5p expression were significantly upregulated in CNS injury. Treatment with their inhibitors reduced inflammatory factor release from microglia, promoted the microglia-derived anti-inflammatory factors, and reduced neuronal death and injured areas (122–124).

### Gut-CNS Axis

Recently, studies have shown that the gut-CNS axis is important in the progression of CNS degeneration and that the gut microbiota has significant effects on microglia in CNS diseases (129–131). Schaedler flora mice, which are colonized with only three bacterial species, acute microbiome-depleted mice, and germ-free mice display obvious microglial abnormalities, including an immature phenotype and altered cell proportions, such as increased branching and terminal points, longer processes, and more segments (129, 132). Fecal microbiota transplantation reduced the activation of microglia and resulted in neuroprotection in both the brain and gut in Parkinson's disease mice, probably through decreased activity of the TLR4/TBK1/NF-κB/TNF-α signaling pathway (133). Tryptophan produced by gut microbiota modulates the aryl hydrocarbon receptor-mediated generation of TGF-α and VEGF-b by microglia to further modulate CNS inflammation (134). The functions of various microbial species and the detailed underlying mechanisms remain uncertain. However, diet and the administration of antibiotics may influence the gut-CNS inflammation axis after SCI. We believe that exploring the relationship between specific microbial strains and microglia is crucial for developing a new treatment for alleviating neuroinflammation and SCI in humans.

## Conclusion and Perspective

Microglia are crucial in inflammation and repair in SCI. After traumatic SCI, microglia are activated by ischemia, anoxia, and damage-associated molecular patterns derived from impaired cells. Microglia play distinct roles in different periods after injury due to shifts in the quantities and proportions of microglia with different phenotypes. Microglia are closely associated with other cells in the spinal cord, and their interactions are more intimate than previously thought. In the acute damage period of SCI, microglia mainly release anti-inflammatory factors, recruit peripheral cells and remove damaged tissues and debris. These effects lead to a reduction in harmful stimuli but will cause more serious damage if the immune reaction is not controlled. In contrast, in the chronic repair period of SCI, microglia mainly limit the spread of inflammation along with glial scarring but also limit the regeneration of neurons and release proinflammatory factors that lead to chronic inflammation and may result in chronic damage.

Although many strategies have been used to target microglia, there are still many new attempts that are worthy of attention. A variety of therapeutic strategies targeting microglia have been proposed in recent years, including chemical drug therapy, physical therapy, biotherapy, biomaterial therapy, and traditional Chinese therapy. Although much progress has been made in the prevention, treatment, and rehabilitation of traumatic SCI, there is still enormous room for improvement. It is vital to accurately modulate the balance of pro-/anti-inflammatory molecules secretion in various periods in SCI. Microglial efferocytosis of damaged cells *via* STAT6/Arg1 regulates microglial polarization, promotes inflammation resolution, and improves stroke outcomes. It is also a novel direction for inflammation resolution and recovery in the injured spinal cord (135).

Microglia are an important component in the processes of spinal cord secondary damage and repair. An accurate and comprehensive understanding of their activation, functions, and mechanisms can be translated into targeted therapy to alleviate secondary damage and promote repair and regeneration in SCI. In future studies, we can focus on the appropriate adjustment of the number and proportion of different microglia to achieve better functional recovery after SCI. It is worth noting that these results come from mice, and there is still a long way to go to translate these results into human clinical practice. However, with the progress made in animal trials and the initiation of clinical trials, we anticipate that the outcomes of patients with SCI will improve in the future.

## Author Contributions

LX, JW, and LW designed the study, acquired the materials, and wrote the manuscript draft. YD and LW edited and revised the manuscript. Y-JZ reviewed and edited the manuscript. All authors contributed to the article and approved the submitted version.

## Funding

This study was supported in part by Zhejiang Public Welfare Technology Application Foundation Experimental Animal Project (No. LGD19H090008) and National Natural Science Foundation of China (Nos. 81972138, 81673777, and 81572229).

## Conflict of Interest

The authors declare that the research was conducted in the absence of any commercial or financial relationships that could be construed as a potential conflict of interest.

## Publisher's Note

All claims expressed in this article are solely those of the authors and do not necessarily represent those of their affiliated organizations, or those of the publisher, the editors and the reviewers. Any product that may be evaluated in this article, or claim that may be made by its manufacturer, is not guaranteed or endorsed by the publisher.
